# A Review of the Management of Bile Leaks

**DOI:** 10.7759/cureus.14937

**Published:** 2021-05-10

**Authors:** Cassidy Gawlik, Mary Carneval

**Affiliations:** 1 General Surgery, Ohio University Heritage College of Osteopathic Medicine, Cleveland, USA; 2 General Surgery, Cleveland Clinic Foundation Euclid Hospital, Cleveland, USA

**Keywords:** post cholecystectomy bile duct injury, classification of bile duct injury, hepatobiliary specialist, strasberg classification, endoscopic retrograde cholangiopancreatography (ercp), economic impact, bile leak management, laparoscopic cholecystectomy, laparoscopic cholecystectomy complication, prevention

## Abstract

Bile leaks can be a complication of abdominal surgeries, specifically trauma to the biliary system during laparoscopic cholecystectomy, and can occur from a variety of sources, commonly a bile duct injury (BDI). Their management involves a multidisciplinary approach and depends on a multitude of factors. This consequence has also led to increased health care costs and morbidity and mortality for patients. Currently, there are no professional society-initiated guidelines that provide surgeons with a clear algorithm for managing bile leaks, as there are for other operative approaches and management in various surgical diseases. Thus, a literature search was performed that surveyed current research on the effective prevention and management of the different types of bile leaks. This review aims to provide all clinicians with an overview of factors to consider in the management of bile leaks and supports referral to a tertiary center with a hepatobiliary specialist.

## Introduction and background

In the United States, approximately 750,000 laparoscopic cholecystectomies are performed annually [1]. This minimally invasive procedure is the accepted technique for the treatment of gallbladder disease due to the low cost, reduced length of hospital stay, quick recovery, and patient satisfaction. Though minimally invasive, there is still a risk for complications. Studies have reported that these occur more frequently in those with risk factors that include age > 65 years, acute cholecystitis, previous cholecystitis, preoperative endoscopic retrograde cholangiopancreatography (ERCP), and conversion to open cholecystectomy [2]. Serious complications were found to occur in 2.6% of cases and include bile duct injury (including bile leaks), bleeding, and bowel injury [3]. Specifically, the incidence of bile duct injury following laparoscopic cholecystectomy has been estimated to be 0.15-0.3% of all cases [4]. This amounts to between 2300 and 3000 bile duct injuries annually, each case involving a 126% cost increase to patients with an average one-year cost of more than $60,000 for those requiring operative intervention [4,5]. Although a bile duct injury (BDI) is uncommon, it is preventable. Surgeons must be aware of the Safe Cholecystectomy Task Force guidelines that promote the safety and success of laparoscopic cholecystectomy while minimizing the risk for bile duct injuries. It is also imperative that when a BDI does occur, clinicians recognize the corresponding symptoms and promptly diagnose and treat the source of the leak to prevent further morbidities. Currently, there is no official clinical practice guideline algorithm for the treatment of bile leaks. The decision on how to treat the leak depends on factors such as the severity and type of injury (defined by the Strasberg classification), the extent of the patient’s illness, presence of stones, and the patient’s risk for complications of sphincterotomy [6]. Studies have also supported successful outcomes with early referral to a tertiary biliary surgery center with a hepatobiliary specialist [7]. In this review, we aim to discuss the factors to consider that should guide decision-making and encourage early referral to a hepatobiliary specialist for the management of bile leaks. 

## Review

The management of bile leaks is multifactorial. It is paramount for surgeons to be familiar with strategies on the prevention of this consequence. In addition, the source of the leak contributes to the clinical presentation of the patient and must thoroughly be evaluated with laboratory and imaging studies. Once these components are assessed, clinicians can then begin to draft a management plan to treat the bile leak and ultimately heal the patient.

Prevention of bile leaks 

Even though bile leaks are infrequent, it is important for surgeons to take the appropriate measures in preventing a bile duct injury. In 2014, the Society of Gastrointestinal and Endoscopic Surgeons (SAGES) formed the Safe Cholecystectomy Task Force intending to create a universal culture of safety around laparoscopic cholecystectomies and to reduce the incidence of biliary injuries [4]. Since bile duct injuries are among the most common serious complication of laparoscopic cholecystectomy, the SAGES task force completed an extensive literature review to formulate safe practice recommendations derived from evidence-based medicine to help prevent this consequence. Strong evidence exists for suggesting that surgeons use the critical view of safety for anatomic identification of the cystic duct and artery [4]. When this anatomical site is identified, it is encouraged that the surgeon takes a momentary pause to confirm positioning before clipping or transecting the ductal or arterial structure. Further, if the critical view of safety cannot be clearly identified, multiple studies have shown evidence to support the use of intraoperative cholangiography for clarification of biliary anatomy. If a surgeon is faced with the uncertainty of anatomy, it is encouraged to have a low threshold when asking for help from another surgeon [4]. When a patient initially presents with acute onset of cholecystitis, the SAGES Cholecystectomy Task Force recommends using risk stratification models, such as the Tokyo Guidelines 18 (TG18) or another effective model, to grade the severity of disease and to guide patient management. Further, it is recommended that surgeons pay consideration to factors that would increase the difficulty of laparoscopic cholecystectomies such as increased age, male sex, emergent cholecystectomy, chronic inflammation, adhesions from previous surgeries, presence of cystic duct stones, and anatomic variations [4]. If the patient is classified as mild acute cholecystitis according to the TG18, it is recommended to perform a laparoscopic cholecystectomy within 72 hours of symptom onset [4]. Moreover, if extensive inflammation is encountered during the procedure that limits identification of the cystic duct and artery, the surgeon should perform a subtotal cholecystectomy either laparoscopically or openly, depending on the surgeon's skill level and comfort. In patients who have acute calculous cholecystitis previously treated with cholecystostomy, it is suggested to allow time for the inflammation to subside before performing an interval cholecystectomy [4]. As far as technique, expert evidence recommends the use of a multi-port laparoscopic cholecystectomy instead of a single port technique. All in all, these above recommendations were drafted by the SAGES Safe Cholecystectomy Task Force to provide surgeons with evidence on surgical techniques and considerations that can help prevent bile duct injuries. Despite following these guidelines, BDI is still a known complication. 

Sources of bile leaks

Bile can leak from a variety of anatomic sites, including the cystic duct remnant, bile ducts of Luschka, and main bile ducts [8]. The most common site is from a cystic duct stump, and this may result from faulty clip applications, slipping of clips, or necrosis of the cystic duct stump proximal to the clip [9]. The Strasberg classification is a useful and easy-to-understand method that categorizes bile injuries from Type A through E based on anatomical location within the biliary system and whether or not there is still communication with the common bile duct [10]. Type A leaks arise from the cystic duct or an accessory duct. Type B injuries involve partial occlusion of the biliary tree, commonly from an atypical right hepatic duct. Type C leaks originate from an aberrant right hepatic duct within the biliary system, but one that has no communication with the common bile duct. Type D leaks are defined as a lateral injury to a major bile duct without loss of connection to the common bile duct. Type E leaks are the most severe with complete transection of the bile duct and include a subclassification of E1-5 that further defines the length of the remnant stump. Bile leaks can result from many different anatomical sites, and identifying the source of the leak is important for selecting the approach for management.

**Figure 1:**
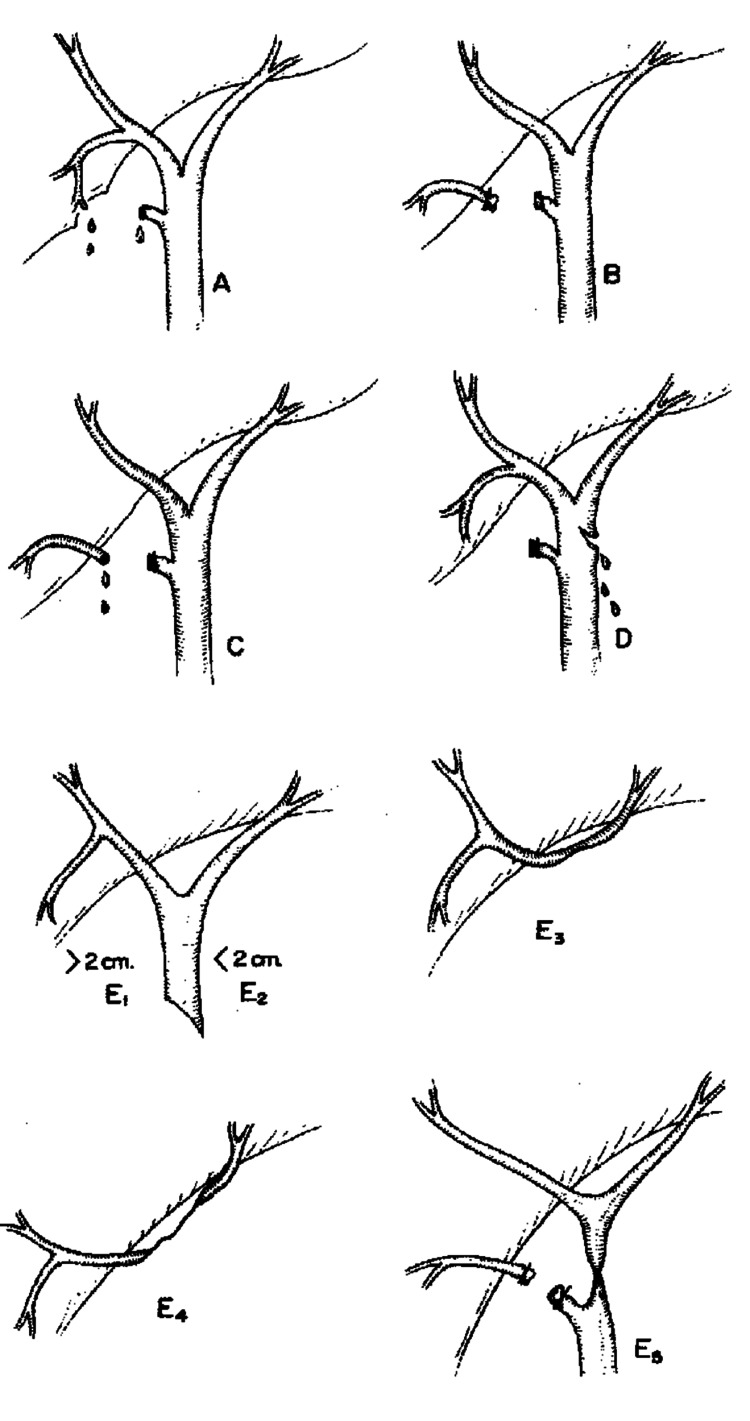
Classification of bile duct injury during laparoscopic cholecystectomy according to Strasberg et al. [11]

Clinical presentation 

When a BDI does occur, most patients have a nonspecific set of symptoms with variability in the timing of their presentation. Some patients may have a delayed presentation, while others can present with life-threatening symptoms of peritonitis, sepsis, cholangitis, or external biliary fistula [12]. Regardless, most patients typically present two to 10 days post-cholecystectomy with fever, abdominal pain, and/or bilious ascites with or without jaundice in addition to bile leaking from incisions. With these symptoms, workup with further labs and imaging should be performed to confirm the diagnosis of a bile leak.

Investigation and diagnostic methods

Biliary injury may be recognized either intraoperatively or more commonly, post-operatively. The use of intraoperative cholangiogram (IOC) has been studied as a method for prevention and early identification of BDI and its use is controversial. Retrospective reviews specific to laparoscopic cholecystectomy and after the development of the critical view of safety have found no added benefit with routine use of IOC in preventing BDI [5]. Thus, it is not recommended as a tool to prevent leaks but rather one to intraoperatively identify when an injury has occurred. Alternatively, patients who present in the post-operative period with signs and symptoms of a bile leak require a thorough evaluation. The diagnostic workup begins with laboratory evaluation and imaging. Serum lab profile, including complete blood count (CBC) and liver function tests (LFTs), can often demonstrate leukocytosis and elevated bilirubin, serum alkaline phosphatase, and gamma-glutamyl transferase, respectively [1]. The diagnostic imaging of choice may begin with a transabdominal ultrasound to determine the presence of a fluid collection. Additional imaging such as CT, hepatobiliary iminodiacetic acid (HIDA) scan (which will confirm a bile leak), and endoscopic retrograde cholangiopancreatography (ERCP) is useful to determine the source of the leak [1]. Magnetic resonance cholangiopancreatography (MRCP) can be done as a noninvasive method to also diagnose the source of the leak. However, the advantage of ERCP is that it is both diagnostic and therapeutic, whereas MRCP can only provide the diagnosis. Once the source has been identified, treatment options are pursued and management strategies frequently involve collaboration with a hepatobiliary surgeon.

Management of the different types of bile leaks 

Currently, there is no worldwide systematic algorithm that guides decision-making in the management of bile leaks. The goal of treatment is to eliminate the transpapillary pressure gradient thereby permitting free flow of bile [1]. This can be achieved through ERCP with sphincterotomy alone, stenting alone, or combination therapy with lower failure rates seen in stenting alone or combination therapy [1]. If necessary, surgical intervention may be pursued. The decision for the type of treatment depends on a variety of factors including the severity of the patient’s presentation and Strasberg's classification of the injury. 

ERCP is recommended as a treatment and has been successful in approximately 90% of cases [9]. Types A, C, and D are typically managed with this modality. Benefits of this procedure include the ability to define the source of the leak, remove any present stones, and sealing the leak. This is most commonly accomplished by means of endoscopic stenting and sphincterotomy or percutaneous transhepatic biliary drainage [13]. As a result, distal bile duct pressure is reduced, allowing for the closure of the site of the leak [14]. For Type A leaks, the patient is followed up in two to four weeks with repeat ERCP to confirm resolution of the leak and removal of the stent. Types C and D leaks require a HIDA scan at two to four weeks post stent insertion, and if the leak has resolved, a repeat ERCP is performed for removal of the stent. Though ERCP has been highly successful, it carries the highest risk among endoscopic procedures and can lead to complications such as pancreatitis, hemorrhage, infection, and intestinal perforation [15]. 

Type B injuries are occlusive and can present years after surgery. As a result of the blockage, hepatic atrophy can occur in correlation with segmental cholestasis. Definitive treatment requires surgical intervention with hepaticojejunostomy and potential segmental resection [1]. 

Similarly, Type E bile leaks are the most severe type and are also managed with surgical intervention. If noticed at the time of laparoscopic cholecystectomy, surgical repair with T-tube drainage at the site of injury can be attempted [1]. If found after surgery, the liver must be decompressed via drainage of hepatic lobes followed by a hepaticojejunostomy [1]. Since Type E leaks involve extensive damage to the biliary system and potentially the liver, invasive measures requiring surgery are necessary to repair the defect. 

An additional question arises; who should be the surgeon that performs the BDI repair, the initial operating surgeon or hepatobiliary specialist? The success of primary repair by the initial operating surgeon is unfavorable for both intraoperative and post-operative injuries. Data evaluating the success of primary repair by the initial operating surgeon shows poor success rates ranging from 17% to 27% [5]. In addition, one review involving medicare beneficiaries found an increase in mortality of 11% when the primary operating surgeon was the one repairing the BDI [5]. These grim statistics are ameliorated when referral to a tertiary center is utilized. It has been found that final success rates of > 90% can be achieved with referral to a tertiary care center [16]. 

All in all, various treatment options have been proven successful based on the different types of bile leaks. Referral to a tertiary center with a hepatobiliary specialist also adds benefit to therapeutic outcomes. This review demonstrates that there is not a current clinical practice guideline available for the management of bile leaks and when to refer to a tertiary center.

Economic and clinical outcomes

The long-term impact of bile leaks can be both clinically and economically substantial. BDI has been found to be a source of increased mortality and poorer quality of life, leading to an 8.8% increase in all-cause mortality [4,5]. In addition, one study found an average cost of more than $60,000 in patients who required operative intervention as a result of a bile duct injury [5]. These are costs that can be prevented. Similarly, one retrospective study analyzed the long-term clinical and economic impact on Type A to D leaks, considered “minor” injuries compared to Type E leak or “major” injuries. They found that although thought to be minor, the rate of bile leaks is highest in Type A to C and that this “minor” group poses a considerable financial burden and long-term complication rate [17]. As a result of these findings, this study drafted a management algorithm that supports the use of a hepatobiliary surgeon when intraoperative recognition of Type A to D leak occurs. 

## Conclusions

A bile leak resulting from laparoscopic cholecystectomy is an uncommon occurrence and can occur from a variety of sources. Although guidelines exist to prevent bile duct injuries, the incidence of bile leaks cannot be eliminated. Prompt identification and treatment are extremely important to prevent increased morbidity, treatment failure, and even death. In patients presenting with symptoms related to a bile leak, laboratory work and imaging with ultrasound, HIDA scan, MRCP, and/or ERCP should be done for proper Strasberg classification and to help guide management. Present day, there is still some debate over the appropriate treatment of bile leakage after laparoscopic cholecystectomy. ERCP is the assumed standard of care with surgical measures reserved for serious cases. By considering the severity and type of bile leak and with the use of an experienced hepatobiliary surgeon, the most beneficial treatment option can be accurately chosen which will improve patient outcomes and decrease the cost of care. With increasing awareness of the different approaches to bile leak management and encouragement of tertiary referral, more physicians will be able to successfully resolve a bile duct injury.
